# Characterizing Mandibular Morphology in Robin Sequence—A 3D Statistical Shape Analysis

**DOI:** 10.1097/SCS.0000000000011326

**Published:** 2025-04-04

**Authors:** Khalid El Ghoul, Eimear O’ Sullivan, Praveen Kumar Guntaka, Gwen G. van Heesch, Koen F.M. Joosten, Bas Pullens, Roman H. Khonsari, Cory M. Resnick, Lara S. van de Lande, Eppo B. Wolvius

**Affiliations:** * Department of Oral and Maxillofacial Surgery, Erasmus Medical Centre, Rotterdam, The Netherlands; † Department of Computing, Imperial College London, London, UK; ‡ Department of Plastic and Oral Surgery, Boston Children’s Hospital, Boston, MA; § Department of Neonatal and Paediatric Intensive Care, Sophia Children’s Hospital, Erasmus Medical Centre; ∥ Department of Otorhinolaryngology, Erasmus Medical Centre, Rotterdam, The Netherlands; ¶ Department of Maxillofacial Surgery and Plastic Surgery, Necker—Enfants Malades Hospital, Assistance Publique—Hopitaux de Paris, Faculté de Medicine, Université Paris Cité, Paris, France

**Keywords:** 3D, mandibular morphology, micrognathia, Robin sequence, shape analysis

## Abstract

**Background:**

Robin sequence (RS) is a congenital condition and constitutes the triad of micrognathia, glossoptosis, and upper airway obstruction. While micrognathia is a cardinal feature of RS, its assessment is largely subjective. The aim of the present study is to describe 3D mandibular morphology in patients with RS and age-related mandibular shape variation compared with an age-matched control group.

**Methods:**

3D reconstructions of the mandible were obtained from CT-imaging of children with isolated (iRS) and nonisolated RS (niRS). Principal Component Analysis was used to describe variation in mandibular morphology. Partial Least Squares and multivariate analysis of variance (MANOVA) were used to compare shape differences between patients with RS and 1:1 age-matched control groups.

**Results:**

A total of 84 patients with iRS and 48 with niRS were included with a mean age of 5.4±8.4 months versus 11.0±13.9 months (*P*-value<0.001). For the iRS and niRS groups, the first principal component primarily constituted allometric shape variation, as a high correlation was noted with age in both groups (Spearman R=0.79). Compared with the control group, both the iRS and niRS mandibles displayed shorter condylar necks, shorter mandibular bodies, and less pronounced, more rounded symphyseal projection (MANOVA, *P*-value<0.001). For both groups, a persistent difference in age-related shape changes along the first shape variable compared with the age-matched control group is observed.

**Conclusions:**

Variation in mandibular morphology in patients with RS for the included age range is primarily due to allometric shape changes. Patients with RS have distinct mandibular morphology relative to age-matched controls. The differences observed in the comparison of age-related shape changes are suggestive of a persistent dysmorphology for patients with iRS and niRS. Future studies will explore the association of mandibular morphology with clinical parameters.

Robin sequence (RS) is a congenital condition and constitutes the triad of micrognathia, glossoptosis, and upper airway obstruction. In an estimated 90% of cases, a cleft palate is also present.^1^ The condition can occur with or without association with a genetic syndrome.^2^ Micrognathia, the primary anomaly in RS, is hypothesized to be the result of either a developmental process, such as genetic factors affecting mandibular bone formation, or deformational factors, including oligohydramnios, abnormal tongue position, and restricted intra-uterine neck extension.^3^ Diagnosis is made by assessment of the neonatal facial profile in combination with observation of glossoptosis and upper airway obstruction.^4^ While micrognathia is a cardinal feature of the Robin sequence, its assessment is largely subjective; no quantitative definition of micrognathia exists.^2,4^

Previous studies have described micrognathia in RS via comparative and longitudinal anthropometric measurements from 2-dimensional (2D) clinical images and radiographs.^5–9^ These studies primarily describe antero-posterior morphologic and positional differences. More recently, mandibular morphology in patients with RS was assessed using 3-dimensional (3D) reconstructions of the mandible from computed tomography (CT) scans.^10–12^ All reported measures utilize traditional morphometrics, including Euclidean distance metrics and derivatives. These are limited in providing a comprehensive description of mandibular morphology.^13,14^

In the field of anthropology, geometric morphometrics is used to quantitatively study shape variations using Cartesian landmark coordinates.^14^ The use of geometric morphometrics is increasingly acknowledged in the study of shape variations for clinical purposes in the craniofacial region.^15^ Mandibular shape variation has recently been described in a normal pediatric population, showcasing the benefit of using geometrics morphometrics in providing a comprehensive, interpretable, and statistically robust method for shape analysis.^16,17^ Only one study has applied geometric morphometrics to 3D reconstructions of the mandible in patients with RS but limited the analysis to the 2D sagittal plane and few anatomic landmarks, without a control group.^18^

The aim of the present study is to assess mandibular morphology in patients with RS between 0 and 4 years of age using 3D morphable models. The purposes of this study were to describe mandibular morphology in patients with RS, and describe age-related shape variation in mandibular morphology in patients with RS compared with an age-matched control group. We hypothesize that the mandible in patients with RS is morphologically distinct from age-matched normal controls and that these characteristics persist with age.

## METHODS

### Study Design and Sample Description

This was a retrospective case-control study of patients diagnosed with RS at the Erasmus Medical Center (EMC) in Rotterdam, the Netherlands, the Boston Children’s Hospital (BCH) in Boston, MA, and Necker–Enfants Malades Hospital (NEMH) in Paris, France. To be included, patients had to: (1) have a clinical diagnosis of RS, ie micrognathia, glossoptosis, and upper airway obstruction necessitating supportive measures or objectified through polysomnography, (2) preoperative CT-scan imaging with slice thickness ≤1.25 mm including the entire mandible, (3) acquired between 0 and 4 years of age. Subjects were separated into isolated (iRS) and non-isolated (niRS) groups. Patients with facial dysostoses, including Treacher Collins, Nager, and Miller syndromes, were excluded because of the asymmetric and severe mandibular deformity observed in these conditions, as this would interfere with the identification of anatomic markings for registration.^11,19^ Patients with other forms of syndromic RS, such as Stickler syndrome, were included in the niRS group. All syndromic diagnoses and identified genetic findings are shown in Supplemental Table 1, Supplemental Digital Content 1, http://links.lww.com/SCS/H668. In case multiple scans were available, the scan with the highest quality was included for each subject.

The normative data used for comparison included unaffected individuals between 0 and 4 years of age with maxillofacial CT scans obtained at NEMH. The variation of normal mandibular morphology for this group was reported previously.^16^ This study received IRB approval at BCH (Protocol #P00023123) and the EMC (MEC 2015-539). Approval at NEMH followed the local protocol.^20^

### Image Processing

**FIGURE 1:**
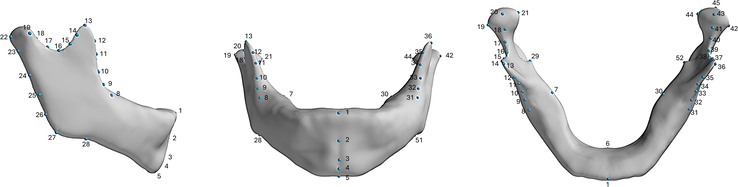
Template mandible consisting of 19,137 vertices and 38,270 triangles with the annotated landmarks shown in blue.

The mandible was segmented from CT scans obtained as DICOM files and converted to a 3D triangular mesh through semi-automated thresholding using Mimics Medical 26.0 (Materialise, Leuven) and Avizo 2022.1 (Visualization Sciences Group, Burlington). All meshes were annotated with validated anatomic landmarks (Fig. 1). All mandibles were then rigidly aligned to a template using the annotated landmarks through a partial Procrustes alignment removing translation and rotation effects but preserving scale. The template was then registered to each sample mandible using the nonrigid Iterative Closest Point (ICP) algorithm in Wrap 3.4 (Faceform, Yerevan). The same set of landmarks was used to guide the registration process. Following registration, the mandibles were aligned once more using all corresponding vertices to achieve an accurate orientation. The template mesh consisted of 19.137 vertices and 38.270 triangles and was created by symmetrizing and remeshing a high-quality median-aged mesh from the normal group data set in MeshMixer (Autodesk, Toronto).^16^ Because of an insufficient number of control samples for a 1:1 ratio of samples with RS to age-matched controls, age-matched synthetic control samples were generated from the normal 3D morphable model using a multivariate Gaussian distribution. The validity of these samples in representing realistic mandibles has been previously described.^16^

### 3D Analysis of Morphology

Principal component analysis (PCA) was used to describe variation in mandibular morphology for each group. To assess differences in shape variation between groups and covariates, Partial Least Squares-Regression (PLSR) or Partial Least Squares-Discriminant Analysis (PLS-DA) was used on the vertex coordinates of registered mandibles. Shape variables most correlated with the direction of variance between groups were extracted. PLS is a supervised method used for classification and dimensionality reduction in machine learning. PLS is similar to PCA, but instead of preserving the greatest variance in its components, it preserves the greatest covariance between class labels and vertex coordinates. Unlike PCA, this allows for a specific comparison of morphology and its covariance with age and associated diagnosis or labels. In this study, the modes of variation are referred to as principal components for PCA and shape variables for PLS. PLSR is used to model age effects and mandibular morphology.

### Statistical Analysis

χ^2^ test was used to compare categorical variables, and Student *t* test or nonparametric tests were used where applicable for continuous variables. The mean mandible for each group was constructed. To test whether the distribution of mandibular morphology in patients with RS was different from age-matched controls, multivariate analysis of variance (MANOVA) was used on extracted shape variables. Differences in age-related shape variation between groups were assessed through Fisher z-transformation on Spearman correlation coefficients of the first shape variable extracted from PLSR and age. Correlation coefficients above 0.70 were considered highly correlated. Data analysis was conducted in Python v3.11. A *P*-value <0.05 was considered significant.

## RESULTS

### Sample Demographics

**FIGURE 2:**
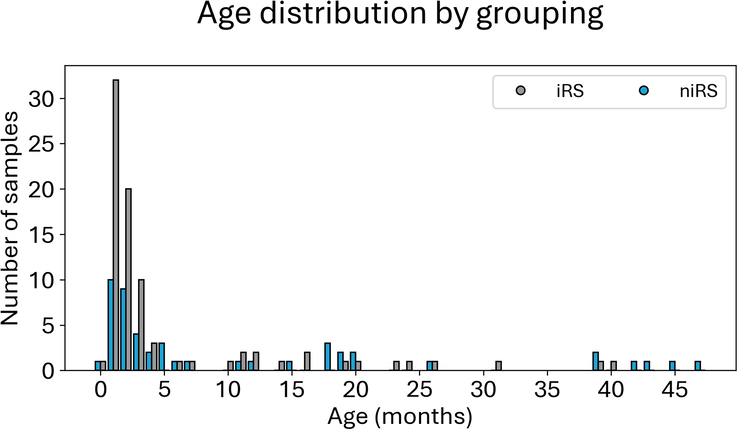
Age distribution in months for the included patients with isolated RS (iRS) and nonisolated RS (niRS). A 1:1 ratio for the age-matched controls was used.

A total of 132 patients with RS (53.0% female) were included (Fig. 2). Of these, 48 (36.4%) were included in the niRS group, among which Stickler syndrome was the most common diagnosis (22.9%, Supplemental Table 1, Supplemental Digital Content 1, http://links.lww.com/SCS/H668). There was a significant difference in age at the time of the included CT imaging between the iRS and niRS groups (5.4±8.4 versus 11.0±13.9 mo, *P*-value <0.001). The control group consisted of 67 age-matched controls (46.0% female). An additional 65 age-matched synthetic samples were generated for the control group to meet the 1:1 sample-to-control ratio.

### Comparison of Mandibular Morphology

#### Variation in Mandibular Morphology in iRS and niRS

**FIGURE 3:**
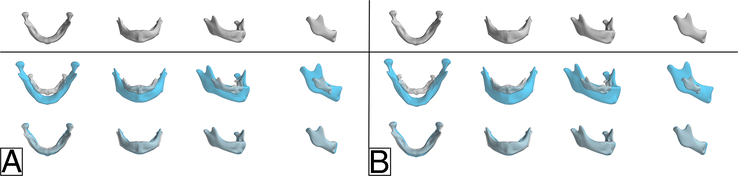
In the top row, the mean mandibular shape for the (A) isolated RS group and (B) non-isolated RS group is shown in gray. Variation in mandibular morphology along the first (middle row) and second (bottom row) principal components is shown. The mandibles in the middle and bottom row present the mandible at +2 SDs (blue) and −2 SDs (gray) along the respective principal component.

The mean mandibular shape and variation in mandibular morphology along the first 2 principal components are depicted in Fig. 3. For the iRS and niRS groups, 82.4% and 84.8% of variance are observed along the first principal component and 9.1% and 9.2% along the second, respectively. The first principal component primarily constituted allometric shape variation as a high and similar correlation was noted with age in both groups (Spearman R=0.79, *P*-value <0.001 and Spearman R=0.79, *P*-value <0.001, respectively). A statistically significant difference was noted in mandibular morphology between the 2 groups (MANOVA, *P*-value <0.013). However, this likely reflected the difference in age between these groups as low correlation coefficients are observed between the 2 groups for all shape variables in PLS-DA (Supplemental Table 2, Supplemental Digital Content 2, http://links.lww.com/SCS/H668).

#### iRS versus Controls

**FIGURE 4:**
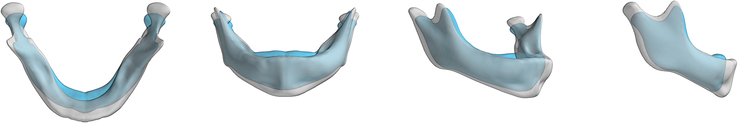
The mean mandibular shape for the isolated RS group (blue) and age-matched control group (gray) from different perspectives.

**FIGURE 5:**
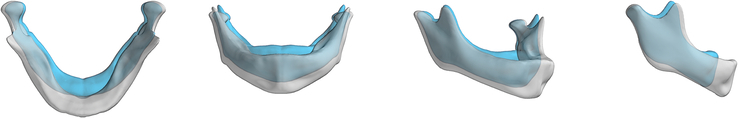
The mean mandibular shape for the isolated RS group (blue) and age-matched control group (gray) rigidly realigned at the condyle from different perspectives for illustrative purposes. The differences in mandibular morphology become more apparent.

**FIGURE 6:**
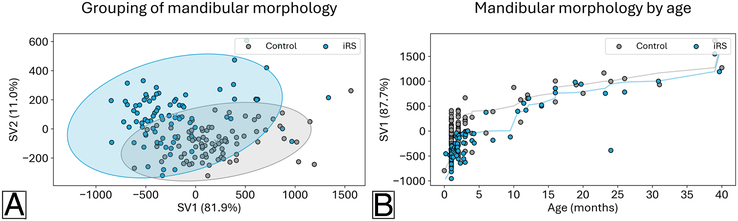
(A) Results from Partial Least Squares-Discriminant Analysis for the isolated RS (iRS) group and the age-matched control group with the first 2 shape variables (SV) shown and associated percentage of variance explained. A larger spread is noted for the iRS group, and some overlap between the 2 groups. (B) Results from combined Partial Least Squares-Regression for the iRS group and age-matched control group with the covariance of age and mandibular morphology along the first shape variable (SV) and associated percentage of variance explained. The line indicates the Spearman correlation by grouping.

A statistically significant difference in mandibular morphology between the iRS group and an age-matched control group was found (MANOVA, *P*-value <0.001) (Fig. 4). Compared with the control group, iRS mandibles displayed shorter condylar necks and shorter mandibular bodies, and less pronounced, more rounded symphyseal projection. The differences in mandibular morphology between the iRS group and age-matched control group become visually more apparent when the mandible is aligned at the condyles (Fig. 5). A separation of the 2 groups is seen in a biplot of the first 2 shape variables from PLSDA with more variation for the iRS group (Fig. 6A).

#### niRS versus Controls

**FIGURE 7:**
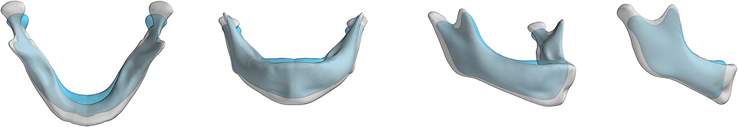
The mean mandible for the nonisolated RS group (blue) and age-matched control group (gray) from different perspectives.

**FIGURE 8:**
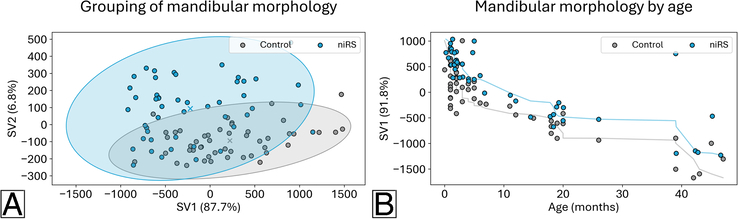
(A) Results from Partial Least Squares-Discriminant Analysis for the non-isolated RS (niRS) group and the age-matched control group with the first 2 shape variables (SV) shown and associated percentage of variance explained. A larger spread is noted for the niRS group, and some overlap between the 2 groups. (B) Results from combined Partial Least Squares-Regression for the niRS group and age-matched control group with the covariance of age and mandibular morphology along the first shape variable (SV) and associated percentage of variance explained. The line indicates the Spearman correlation by grouping.

A similar difference was noted between the niRS group and age-matched controls (Fig. 7) and demonstrates similar shape differences as noted in the iRS comparison (MANOVA, *P*-value <0.001). Specifically, when compared with control mandibles, niRS samples had a shorter condylar neck and mandibular body. The biplot from PLSDA for the 2 groups also shows a larger spread for the niRS group than the age-matched controls and distinct clustering of the 2 groups (Fig. 8A).

### Comparison of Age-related Shape Changes

The age-related shape changes in mandibular morphology along the first shape variable for the iRS group and the age-matched control group are shown in (Supplemental video 1, Supplemental Digital Content 3, http://links.lww.com/SCS/H808). The explained variance included was 88.7% and 89.2%, respectively (Supplemental Table 3, Supplemental Digital Content 1, http://links.lww.com/SCS/H668). Persistent differences between the 2 groups are noted along the included age range. Specifically, for the iRS group, the condylar elongation, symphyseal projection, anterior body elongation, and decrease of the gonial angle with age are limited relative to the control group. In a comparison of age effects on mandibular morphology, a divergence is noted between the iRS group and the age-matched controls (Fig. 6B, Spearman R=0.79 versus Spearman R=0.58, *P*-value=0.007). In (Supplemental video 2, Supplemental Digital Content 4, http://links.lww.com/SCS/H809), a similar persistence in age-related dysmorphology is noted for the niRS group and the control group, with 90.3% and 94.5% of explained variance included along the first shape variable, respectively (Supplemental Table 4, Supplemental Digital Content 1, http://links.lww.com/SCS/H668). Unlike the age effects observed in the iRS group, no statistical difference between the niRS group and controls was noted (Fig. 8B, Spearman R=−0.80 versus Spearman R=−0.76, *P*-value=0.730).

## DISCUSSION

The aim of this study was to describe mandibular morphology in patients with RS through the use of 3D geometric morphometric means, a comprehensive and robust method to analyze shape variation and provide detailed visualizations of persevering spatial relationships.

Significant differences were found in mandibular morphology between iRS and niRS and age-matched controls. In addition to distinct morphologic features, a larger degree of shape variation was noted for both groups compared with controls. This highlights the heterogeneity observed in patients with RS. Furthermore, a high correlation with age was noted for morphologic variation. This can be attributed to allometric shape variation, ie shape differences inherent to differences in size. A persistent dysmorphology in mandibular shape along the included age range was observed for both RS groups compared with their respective control group.

The mean mandibular shape for the RS mandibles had shorter condylar necks, rami and mandibular bodies, more rounded symphyses, and wider gonial angles. These findings are consistent with those previously reported. Susarla et al^10^ compared samples with both iRS and niRS against age-matched controls and utilized traditional measurements for comparative purposes. The samples in their study had a similar demographic distribution to the current study and resemble the presented mean mandibular shape for the RS patients. The same is observed in the findings presented by Volk et al.^21^ However, that study reported a more acute gonial angle in patients with RS compared with their control group. This is not described as such in a systematic review of mandibular morphologic changes of the mandible in patients with RS after mandibular distraction osteogenesis (MDO).^22^ Instead, similar characteristics are described in the current study, including a more obtuse gonial angle.

The persistent age-related differences in mandibular shape between the iRS and niRS groups and their respective control groups indicate that the mandible does not normalize with growth for the included age range. The observed persistence in dysmorphology of the mandible is concordant with previous studies of 3D mandibular morphology. Susarla et al^23^ also reported a persistently dysmorphic mandible of age-matched patients with RS who did not undergo MDO compared with those who had at the end of the consolidation phase. In Wiechers et al,^9^ report is made of an improved facial profile in patients with isolated RS treated with a functional appliance with a velopharyngeal extension during the first year of life. While this was analyzed on 3D soft tissue measures, a persistent temporal difference in the jaw index used in their study compared with a control group is also noted. A similar finding of 2D measurements was reported by Van der Plas et al^24^ and Figuero et al^6^ using lateral profile pictures and radiographic cephalograms, respectively.

Of note, the current study made a distinction between patients with iRS and niRS, whereas other studies often considered these populations as one study group.^11,25^ In Duarte et al,^26^ significant differences were noted between the iRS and niRS mandible. However, they also included patients with Treacher Collins syndrome in their syndromic group, which can contribute to the observation of a shorter ramus length and larger gonial angle in the syndromic group compared with their isolated group. While mandibular morphology was also significantly different between iRS and niRS groups in our study, the age distribution also differed, which precludes conclusive remarks as mandibular morphology is strongly correlated with age. In an examination of age-related shape changes for the iRS group and niRS group in our study, a similar evolution of mandibular morphology is observed.

The findings presented in the current study are relevant in various aspects pertaining to the diagnosis and treatment of patients with RS. The mandibular shape differences described between patients with RS and the age-matched control group provide an appreciation for its role in airway obstruction. In Prescher et al,^22^ the airway obstruction in patients with RS is largely attributed to mandibular shape anomalies anterior to the gonial angle, ie, a shorter mandibular body and rounded symphysis. However, the observed differences presented in the current study could be suggestive of a vertical component, ie, the shorter condyle and lesser extended ramus and the associated cranial positioning of the mandibular body. This leads to a relatively retruded and elevated floor of the mouth. Regardless of its potential association with airway obstruction, this observation could contribute to a limited volume in the oropharynx.

The variation in mandibular morphology observed in patients with iRS and niRS in our study can be assessed against various clinical parameters, for example, severity of airway obstruction, feeding impairment, and growth or underlying genotype. Potential associations can aid the characterization of patients with RS by mandibular morphology and tailor treatment for individual patients, for example, through prognostic characterization of mandibular growth. Previous studies have suggested differences in traditional mandibular measures between patients with RS treated with MDO as opposed to those that were managed nonoperatively.^23^ Gao et al^18^ identified 3 classes of patients with iRS based on mandibular morphology using unsupervised clustering. However, they only used a 2D sagittal representation of the mandible and did not include control samples. The methods adopted in our study simultaneously allow for a comprehensive 3D assessment of mandibular morphology and interpretable visualization of morphologic variation preserving spatial relationships. In addition, both an unsupervised classification of mandibular morphology as well as a comprehensive analysis of covariance of mandibular morphology with clinical parameters can be made. This provides grounds to further investigate the association of mandibular morphology with various clinical measures.

As the current study is a retrospective case-control study, there are some limitations to this work. First, there is an inherent selection bias due to the sole inclusion of patients who had CT imaging. A CT-scan is typically acquired for the planning of MDO in this patient population. As such, this may lead to the selection of more severely affected patients and the omission of patients with milder phenotypes. In addition, there was no systematic documentation of clinical data that was taken at the time of each scan to be used for clinical correlation of mandibular morphology, for example, same-day polysomnography. Furthermore, the scans were taken at different time points for the included patients, which limits the assessment of covariation between mandibular morphology and age to a cross-sectional analysis. Nonetheless, the current sample is among the largest reported in the literature and employs state-of-the-art methods to analyze mandibular morphology in patients with iRS and niRS.

In conclusion, patients with RS have distinct mandibular morphology relative to age-matched controls. Variation in mandibular morphology for the included age range is primarily due to allometric shape changes. The shape differences observed in the comparison of age-related shape changes are suggestive of persistent dysmorphology with growth for patients with iRS and niRS. The mandible does not seem to normalize over time for patients in the included age range. In addition, there is a larger spread in variation in mandibular morphology for the iRS and niRS compared with the control groups. This is in accordance with the clinical heterogeneity observed in patients with RS. The presented findings contribute to our understanding of the pathophysiology in RS and can aid treatment planning. Future studies will explore the association of mandibular morphology with various clinical parameters.

## Supplementary Material

SUPPLEMENTARY MATERIAL
