# Supplementary material for: Characterizing Mandibular Morphology in Robin Sequence—A 3D Statistical Shape Analysis
Source: J Craniofac Surg. 2025 Apr 4;36(8):2976–81. doi: 10.1097/SCS.0000000000011326 (PMC12537024; doi:10.1097/SCS.0000000000011326)
Supplement: SUPPLEMENTARY MATERIAL [file scs-36-02976-s001.docx]

**Tables**

**Supplemental Table 1**. Overview of syndromic and genetic variants in the non-isolated RS group.

| Syndrome | Count (%) |
| --- | --- |
| Stickler syndrome | 11 (22.9%) |
| Undefined | 7 (14.6%) |
| Kabuki syndrome | 4 (8.3%) |
| 16p11.2 deletion syndrome | 2 (4.2%) |
| 18q deletion syndrome | 2 (4.2%) |
| 22q11 deletion syndrome | 2 (4.2%) |
| Fetal Alcohol syndrome | 2 (4.2%) |
| Moebius syndrome | 2 (4.2%) |
| 2q33.1 deletion syndrome | 2 (4.2%) |
| Other* | 14 (29.2%) |

*Other included one sample of each of the following conditions 14q partial trisomy syndrome, 16p12.2 deletion syndrome, 17p13 deletion syndrome, translocation t(3;18), Klinefelter syndrome, Andersen Tawil syndrome, Baraitser-Winter syndrome, Loeys-Dietz syndrome, Hunter syndrome, neonatal abstinence syndrome, Rubinstein Taybi syndrome, trisomy 9, U2AF2-mutation, WTX-mutation. **Abbreviations**: Robin sequence (RS).

**Supplemental Table 2.** The correlation coefficients and percentage of explained variance for the first ten shape variables extracted from the PLS-DA model in comparing the isolated RS and non-isolated RS group.

| **Shape variable** | **Spearman's R** | **P-value** | **Explained variance** |
| --- | --- | --- | --- |
| 1 | 0.16 | 0.069 | 90.5% |
| 2 | -0.14 | 0.108 | 2.7% |
| 3 | -0.27 | <0.001 | 1.7% |
| 4 | 0.21 | 0.016 | 1.0% |
| 5 | -0.30 | <0.001 | 0.3% |
| 6 | 0.19 | 0.026 | 0.9% |
| 7 | -0.31 | <0.001 | 0.2% |
| 8 | -0.24 | 0.007 | 0.3% |
| 9 | -0.24 | 0.005 | 0.2% |
| 10 | -0.26 | 0.003 | 0.1% |

**Abbreviations**: Robin sequence (RS), Partial Least Sqaures – Discriminant Analysis (PLS-DA).

**Supplemental Table 3.** The correlation coefficients and percentage of explained variance for the first ten shape variables extracted from the PLSR model for the isolated RS group and control group.

|  | **iRS group** | | | **Control group** | | |
| --- | --- | --- | --- | --- | --- | --- |
| **Shape variable** | **Spearman's R** | **P-value** | **Explained variance** | **Spearman's R** | **P-value** | **Explained variance** |
| 1 | 0.79 | <0.001 | 88.7% | 0.58 | <0.001 | 89.2% |
| 2 | 0.02 | 0.860 | 3.3% | 0.32 | 0.003 | 2.1% |
| 3 | 0.16 | 0.157 | 2.8% | 0.28 | 0.010 | 0.9% |
| 4 | 0.14 | 0.195 | 1.1% | -0.02 | 0.830 | 2.3% |
| 5 | -0.04 | 0.721 | 0.7% | -0.05 | 0.673 | 1.1% |
| 6 | 0.25 | 0.022 | 0.5% | -0.23 | 0.038 | 0.5% |
| 7 | 0.18 | 0.103 | 0.2% | -0.03 | 0.789 | 0.3% |
| 8 | 0.11 | 0.340 | 0.1% | 0.01 | 0.919 | 0.4% |
| 9 | -0.16 | 0.157 | 0.2% | 0.00 | 0.989 | 0.2% |
| 10 | -0.08 | 0.461 | 0.1% | 0.05 | 0.654 | 0.2% |

**Abbreviations**: isolated Robin sequence (iRS), Partial Least Sqaures – Regression (PLSR).

**Supplemental Table 4.** The correlation coefficients and percentage of explained variance for the first ten shape variables extracted from the PLSR model for the non-isolated RS group and control group.

|  | **niRS group** | | | **Control group** | | |
| --- | --- | --- | --- | --- | --- | --- |
| **Shape variable** | **Spearman's R** | **P-value** | **Explained variance** | **Spearman's R** | **P-value** | **Explained variance** |
| 1 | 0.80 | <0.001 | 90.3% | 0.76 | <0.001 | 94.5% |
| 2 | 0.15 | 0.313 | 3.9% | -0.28 | 0.053 | 1.1% |
| 3 | -0.06 | 0.663 | 1.0% | -0.06 | 0.680 | 0.6% |
| 4 | 0.07 | 0.637 | 0.8% | -0.06 | 0.675 | 0.3% |
| 5 | 0.17 | 0.241 | 0.3% | 0.14 | 0.352 | 0.7% |
| 6 | -0.12 | 0.400 | 0.4% | -0.05 | 0.732 | 0.8% |
| 7 | -0.01 | 0.955 | 0.7% | 0.21 | 0.148 | 0.3% |
| 8 | -0.04 | 0.809 | 0.4% | -0.12 | 0.406 | 0.1% |
| 9 | 0.08 | 0.600 | 0.3% | 0.19 | 0.185 | 0.1% |
| 10 | 0.07 | 0.637 | 0.2% | 0.07 | 0.623 | 0.1% |

**Abbreviations**: non-isolated Robin sequence (niRS), Partial Least Sqaures – Regression (PLSR).
